# Presenilin-1 regulates induction of hypoxia inducible factor-1α: altered activation by a mutation associated with familial Alzheimer's disease

**DOI:** 10.1186/1750-1326-5-38

**Published:** 2010-09-23

**Authors:** Rita De Gasperi, Miguel A Gama Sosa, Stella Dracheva, Gregory A Elder

**Affiliations:** 1 Research and Development, James J Peters Department of Veterans Affairs Medical Center, Bronx, NY 10468, USA; 2 Neurology Service, James J Peters Department of Veterans Affairs Medical Center, Bronx, NY 10468, USA; 3 Department of Psychiatry, Mount Sinai School of Medicine, New York, NY 10029, USA; 4 Department of Neurology, Mount Sinai School of Medicine, New York, NY 10029, USA

## Abstract

**Background:**

Mutations in presenilin-1 (*Psen1*) cause familial Alzheimer's disease (FAD). Both hypoxia and ischemia have been implicated in the pathological cascade that leads to amyloid deposition in AD. Here we investigated whether Psen1 might regulate hypoxic responses by modulating induction of the transcription factor hypoxia inducible factor 1-α (HIF-1α).

**Results:**

In fibroblasts that lack Psen1 induction of HIF-1α was impaired in response to the hypoxia mimetic cobalt chloride, as well as was induction by insulin and calcium chelation. Reintroduction of human Psen1 using a lentiviral vector partially rescued the responsiveness of *Psen1-/- *fibroblasts to cobalt chloride induction. HIF-1α induction did not require Psen1's associated γ-secretase activity. In addition, the failure of insulin to induce HIF-1α was not explicable on the basis of failed activation of the phosphatidylinositol 3-kinase (PI3K/Akt) pathway which activated normally in *Psen1-/- *fibroblasts. Rather we found that basal levels of HIF-1α were lower in *Psen1-/- *fibroblasts and that the basis for lower constitutive levels of HIF-1α was best explained by accelerated HIF-1α degradation. We further found that Psen1 and HIF-1α physically interact suggesting that Psen1 may protect HIF-1α from degradation through the proteasome. In fibroblasts harboring the M146V Psen1 FAD mutation on a mouse Psen1 null background, metabolic induction of HIF-1α by insulin was impaired but not hypoxic induction by cobalt chloride. Unlike *Psen1-/- *fibroblasts, basal levels of HIF-1α were normal in FAD mutant fibroblasts but activation of the insulin-receptor pathway was impaired. Interestingly, in *Psen1-/- *primary neuronal cultures HIF-1α was induced normally in response to cobalt chloride but insulin induction of HIF-1α was impaired even though activation of the PI3K/Akt pathway by insulin proceeded normally in *Psen1-/- *neuronal cultures. Basal levels of HIF-1α were not significantly different in *Psen1-/- *neurons and HIF-1α levels were normal in *Psen1-/- *embryos.

**Conclusions:**

Collectively these studies show that Psen1 regulates induction of HIF-1α although they indicate that cell type specific differences exist in the effect of Psen1 on induction. They also show that the M146V Psen1 FAD mutation impairs metabolic induction of HIF-1α, an observation that may have pathophysiological significance for AD.

## Background

Alzheimer's disease (AD) is a neurodegenerative disorder characterized clinically by progressive loss of memory along with other cognitive skills and pathologically by accumulation of amyloid plaques and neurofibrillary tangles [1]. While most cases occur sporadically some are inherited in an autosomal dominant fashion. Familial cases (FAD) exhibit similar clinical and pathological features as the sporadic disease but have a generally earlier age of onset [2].

The presenilin-1 gene (*Psen1*) was discovered because mutations in it and a homologous gene *presenilin-2 *(*Psen2*) cause FAD [2]. To date more than 170 different mutations in *Psen1 *have been associated with FAD [3]. Mutations in *Psen1 *are the most commonly recognized cause of early onset FAD accounting for probably 50% of all autosomal dominant cases [4]. Mutations in *Psen2 *are a less common cause of FAD [2].

The pathophysiological cascade in AD includes deposition of β-amyloid (Aβ) in compact and diffuse plaques as well as production of oligomeric forms of Aβ that are currently thought to be the most toxic species [5]. The factors that trigger the amyloid cascade remain incompletely understood. Recently there has been interest in the role that vascular disease might play in this process [6]. Indeed an array of epidemiologic evidence supports an association between vascular disease and its risk factors with cognitive impairment and AD [6] and postmortem studies have shown that AD is complicated by vascular pathology in about one-third of cases [7].

These observations could be explained by viewing vascular disease as a co-morbidity that acts in an additive fashion with AD pathology to exacerbate ongoing cognitive decline. Alternatively vascular disease might lead to hypoxia/ischemia that might drive AD pathology itself. Supporting the latter hypothesis both hypoxia and ischemia increase amyloid precursor protein (APP) expression as well as shunt APP processing towards Aβ production [8-13]. Hypoxia also decreases expression of the α-secretase, ADAM-10 [11] while increasing expression of the principal β-secretase, BACE1 [14,15].

Thus aberrant or inefficient hypoxic responses in brain might contribute to AD initiation or progression. The brain's response to hypoxia involves a multiplicity of pathways that as in other tissues includes induction of a set of genes regulated by the transcription factor hypoxia inducible factor-1 (HIF-1)[16,17]. HIF-1 is a dimer that consists of α and β subunits. Levels of HIF-1α are regulated by oxygen availability while HIF-1β subunits are constitutively expressed with the level of HIF-1 transcriptional activity regulated primarily by levels of HIF-1α. HIF-1 is a key transcriptional regulator of a broad range of cellular genes that mediate responses to hypoxia. Although originally discovered as an hypoxia induced factor, HIF-1α also increases in response to stimulation by a number of metabolic/growth factor signaling pathways including insulin and insulin like growth factor-1 (IGF-1)[18-23]. Interestingly both Psen1 and Psen2 are induced by hypoxia [24-26] and Psen1 has been suggested to regulate signaling through a number of growth factor related pathways [27-29]. In addition HIF-1α mRNA was found reduced in a hypomorphic *Psen1 *mouse mutant [30,31]. Here we investigated whether Psen1 might regulate hypoxic responses and show that Psen1 influences both hypoxic and metabolic/growth factor induction of HIF-1α in cells that lack Psen1 although the effects vary in a cell type dependent manner. We also show that a *Psen1 *FAD mutation alters metabolic induction of HIF-1α.

## Results

### Impaired induction of HIF-1α in fibroblasts lacking Psen1

**Figure 1:**
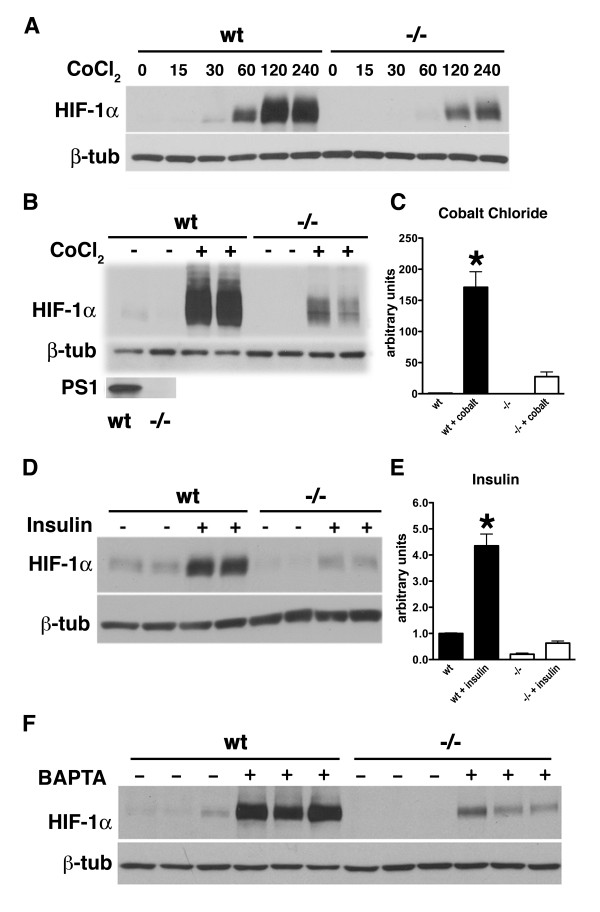
**Impaired induction of HIF-1α in *Psen1-/- *immortalized mouse embryonic fibroblasts**. In panel A, the time course of HIF-1α induction in *Psen1+/+ *(wt) and *Psen1-/- *(-/-) immortalized mouse embryonic fibroblasts is shown. Cultures were treated with 100 μm cobalt chloride for the indicated times (min). Lysates were prepared and Western blotting was performed using an anti-HIF-1α antibody. The lower panel shows the blot reprobed for β-tubulin. A representative blot is shown from experiments that were performed multiple times. In panels B and C, *Psen1+/+ *(wt) and *Psen1-/- *(-/-) cultures were treated with 100 μm cobalt chloride for 4 hours and analyzed for HIF-1α as in (A). The lowest panel in B shows representative samples of wt and -/- fibroblasts probed with the Psen1 antibody 33B10 to confirm the lack of detectible Psen1 expression in *Psen1-/- *fibroblasts. Panel C shows quantitation of the levels of HIF-1α in data derived from four independent experiments. In panels D and E cultures were serum starved overnight and then treated with insulin for 4 hours after which lysates were prepared and Western blotting performed as in (A). Panel E shows quantitation of the experiment shown in (D). In panel F, *Psen1+/+ *and *Psen1-/- *fibroblasts were treated with BAPTA-AM for 1.5 hours. Results from an experiment performed in triplicate is shown. The lower panel shows the blot reprobed for β-tubulin. All panels show representative blots from experiments that were performed multiple times. Western blots for HIF-1α were performed using a rabbit polyclonal antibody. The quantitative results are expressed as the ratio of HIF-1α to β-tubulin and are presented as + SEM. Asterisks in panels C and E indicate values that are different from the corresponding untreated cells (p < 0.05, unpaired t-tests). Other statistical comparisons are discussed in the text.

Psen1 and Psen2 are induced by hypoxia [24-26] suggesting that presenilins may play a role in cellular adaptation to hypoxia. HIF-1α is induced by hypoxia and is a key transcriptional regulator of a broad range of cellular genes that mediate cellular responses to hypoxia [16,17]. To determine whether Psen1 might affect HIF-1α induction we treated *Psen1+/+ *and *Psen1-/- *immortalized mouse embryonic fibroblasts with CoCl_2_. Cobalt chloride mimics the hypoxic activation of HIF-1α by interfering with binding of the iron group that along with oxygen is required for activity of the prolyl hydroxylases (PHDs), that target HIF-1α for proteasomal degradation [32]. When the time course of induction was analyzed it was found that HIF-1α induction was maximal between 2-4 hours after treatment of *Psen1 +/+ *cells, while *Psen1-/- *cells showed considerably less HIF-1α at all time points (Figure 1A). At 4 hours after treatment HIF-1α levels were approximately seven-fold higher in *Psen1+/+ *fibroblasts compared to *Psen1-/- *fibroblasts (p = 0.02; Figure 1B, C).

Besides its effects on hypoxia-induced responses HIF-1α also mediates metabolic and growth factor signaling [33]. For example both IGF-1 and insulin stimulate HIF-1α accumulation [21,34]. To test whether Psen1 might also influence non-hypoxic signaling we stimulated *Psen1+/+ *and *Psen1-/- *fibroblasts with insulin. HIF-1α increased over four-fold in *Psen1+/+ *fibroblasts (p = 0.02; Figure 1D, E). By contrast HIF-1α elevated only slightly in *Psen1-/- *fibroblasts (p = 0.04) and induced levels were more than eight-fold lower in *Psen1-/- *cells than in *Psen1+/+ *cells (p = 0.0002).

HIF-1α is also induced when cellular calcium levels are lowered [35]. To test whether lack of Psen1 might affect induction of HIF-1α in response to altered calcium levels we treated *Psen1+/+ *and *Psen1-/- *fibroblasts with the calcium chelator BAPTA. As shown in figure 1F, *Psen1-/- *fibroblasts activated HIF-1α less than *Psen1+/+ *fibroblasts following calcium chelation.

**Figure 2:**
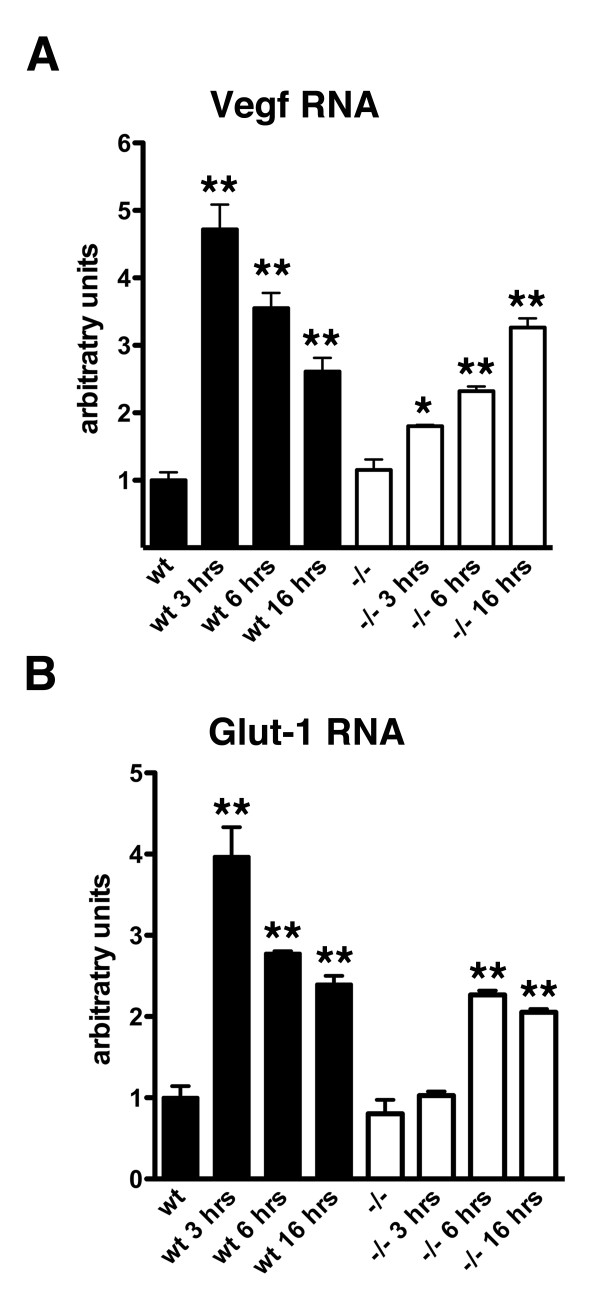
**Reduced activation of HIF-1α downstream target genes in *Psen1-/- *fibroblasts treated with cobalt chloride**. *Psen1+/+ *(wt) or *Psen1-/- *fibroblasts were treated with cobalt chloride for the indicated times (hours). Levels of Vegf (A) and Glut-1 (B) RNA were determined by qPCR (n = 9 cultures per group untreated; n = 3 per group treated). (*) indicates p < 0.05 and (**) p < 0.01 vs. untreated control by Dunnett's post-test. Statistical comparisons between groups are discussed further in the text.

HIF-1α has many downstream targets [17]. To determine whether the reduced induction of HIF-1α in *Psen1-/- *fibroblasts affected activation of HIF-1α target genes, we examined activation of the HIF-1α targets, vascular endothelial growth factor (Vegf) and the glucose transporter-1 (Glut-1) [17] by qPCR. At baseline neither Vegf nor Glut-1 RNA differed between *Psen1+/+ *and *Psen1-/- *cells (p = 0.43 Vegf; p = 0.38 Glut-1, unpaired t-tests). As shown in Figure 2A, following treatment of *Psen1+/+ *cells with cobalt chloride, Vegf RNA was elevated over four-fold at 3 hours post-treatment falling at 6 and 16 hours although remaining elevated compared to baseline (F_3, 14 _= 74.46, p < 0.0001; p < 0.01 all treated groups vs. baseline, Dunnett's test). By contrast, while Vegf RNA was elevated in *Psen1-/- *fibroblasts after treatment with cobalt chloride (F_3, 14 _= 27.59, p < 0.0001), the response was blunted rising to only≈ 1.8 fold at 3 hours (p < 0.05, Dunnett's test), ≈ 2.4 fold at six hours (p < 0.01) and ≈ 3.3 fold at 16 hours (p < 0.01). At 3 hours post-treatment Vegf RNA was approximately 2.5-fold higher in *Psen1+/+ *compared to *Psen1-/- *cells (p = 0.01, unpaired t-test with Welch correction). Interestingly at 16 hours post-treatment levels in *Psen1-/- *were not significantly different from those in *Psen1+/+ *cells (p = 0.054, unpaired t-test).

Very similar results were seen with Glut-1 (Figure 2B). In wild type cells Glut-1 RNA became maximally elevated at 3 hours and continued to be increased over baseline at six and 16 hours (F_3, 14 _= 45.14, p < 0.0001, p < 0.01 all treated groups vs. control, Dunnett's test). By contrast while increasing in *Psen1-/- *fibroblasts (F_3, 14 _= 15.12, p = 0.0001), a blunted response was seen with no change in Glut-1 RNA at 3 hours post-treatment and a maximal induction that was delayed and was only approximately 60% of that seen in *Psen1+/+ *cells at 3 hours. Thus while *Psen1-/- *fibroblasts are not unresponsive to cobalt chloride they clearly show a blunted response with only prolonged exposures producing the levels of induction seen in wild type cells at shorter times, which is consistent with the lower levels of HIF-1α induced by this treatment.

**Figure 3:**
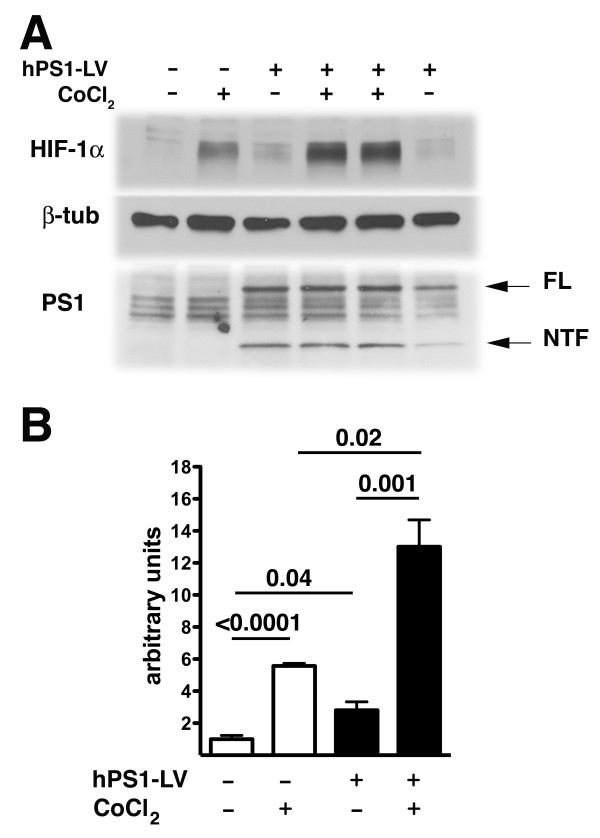
**Reintroduction of Psen1 into *Psen1-/- *fibroblasts**. *Psen1-/- *fibroblasts were infected with lentiviruses carrying the human Psen1 cDNA (hPS1-LV) for 24 hours and grown in complete medium for 48 hours. The infected cells were then treated for 4 hours with 100 μM cobalt chloride along with non-infected *Psen1-/- *cells grown in parallel. The cells were lysed and analyzed for HIF-1α induction by Western blot using anti HIF-1α polyclonal antibodies. Blots were reprobed for anti β-tubulin as a loading control. Psen1 expression was analyzed using the NT.1 mouse monoclonal antibody. FL: full length, NTF: N-terminal fragment. A representative blot is shown in panel A. Panel B shows quantitation of the HIF-1α response (n = 3 samples per group non-infected; n = 4 infected). P values (unpaired t-tests) are indicated for selected comparisons which are discussed further in the text.

To determine whether reintroduction of Psen1 could improve HIF-1α responsiveness in *Psen1-/- *fibroblasts, cells were infected with Psen1-expressing lentiviruses for 48 hours and subsequently treated for 4 hours with cobalt chloride. As shown in Figure 3, reintroduction of Psen1 resulted in an approximately 2.8 fold increase in basal HIF-1α levels in *Psen1-/- *cells (p = 0.04, unpaired t-test) and following treatment with cobalt chloride there was an increase in HIF-1α in both non-infected as well as infected cells (p < 0.0001 non-infected; p = 0.001 infected). However there was a more than two-fold increase in HIF-1α levels in infected as compared to non-infected cells that were grown and treated in parallel (p = 0.02).

**Figure 4:**
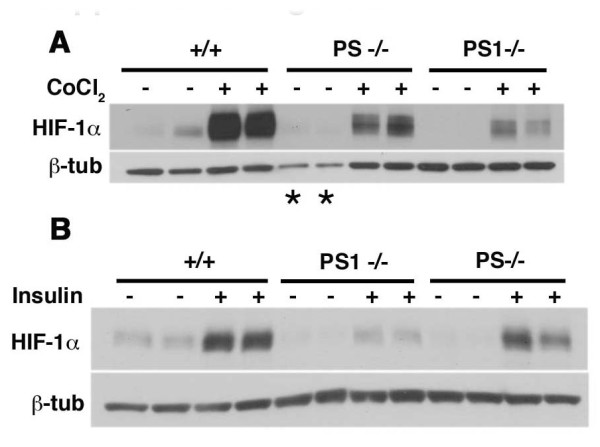
**Impaired induction of HIF-1α in immortalized mouse embryonic fibroblasts lacking both Psen1 and Psen2**. *Wild type*, *Psen1-/- *(PS1-/-) and *Psen1/Psen2-/- *(PS-/-) immortalized fibroblasts were treated with 100 μm cobalt chloride for 4 hours (A) or 2.4 μg/ml of insulin for 6 hours (B). Lysates were prepared and Western blotting was performed using an anti HIF-1α antibody. Lower panels show blots reprobed for β-tubulin. Asterisks (*) indicate lanes that were under loaded based on β-tubulin levels. Note the lowered induction of HIF-1α in the cells lacking both presenilins. Representative blots are shown from experiments that were performed multiple times.

We have also seen less HIF-1α induction in fibroblast cell lines lacking both presenilins (*Psen-/-*) in response to cobalt chloride and insulin (Figure 4). Therefore these results as well as the increased responsiveness of *Psen1-/- *fibroblasts following reintroduction of Psen1 argue against the possibility that the observations are the result of a purely clonal effect. Rather these results argue that fibroblasts lacking Psen1 are hyporesponsive to a range of HIF-1α inducers and that this hypoactivation has functional consequences for the induction of HIF-1α target genes.

### HIF-1α induction does not require γ-secretase activity

**Figure 5:**
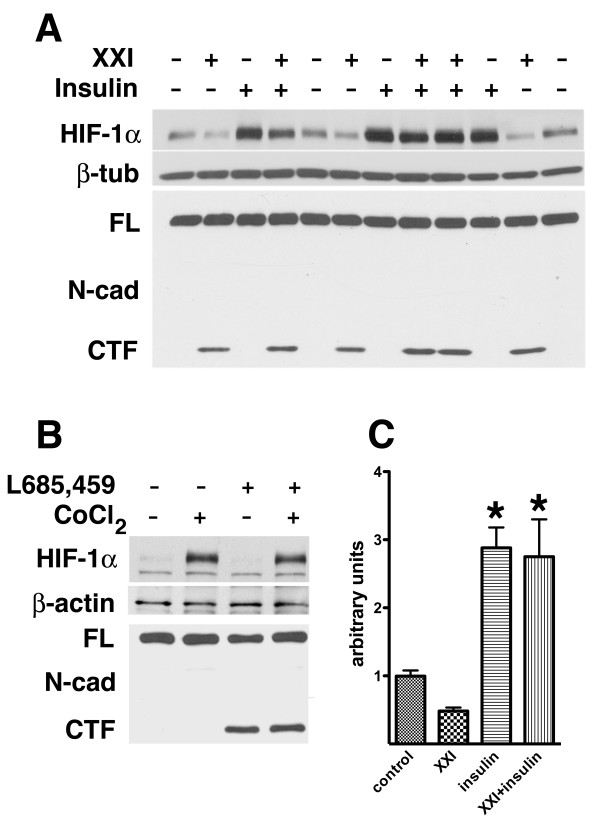
**γ-secretase activity is not needed for insulin or cobalt induction of HIF-1α**. *Psen1+/+ *immortalized fibroblasts (A) were treated overnight with the γ-secretase inhibitor XXI and then stimulated with insulin for 6 hours. Western blotting for HIF-1α was performed (upper panel) followed by reprobing of the blot for β-tubulin (middle panel). The same samples were then reblotted and probed for N-cadherin (lower panel). A band for the full length N-cadherin (FL) is indicated. A band that corresponds to the N-cadherin CTF is visible in the γ-secretase inhibitor treated lanes. In panel B, *Psen1+/+ *fibroblasts were treated overnight with the γ-secretase inhibitor L-685,458 and then stimulated with cobalt chloride for 6 hours. The samples were blotted and probed for HIF-1α (upper panel) and β-actin (middle panel) and then reblotted and probed for N-cadherin (lower panel). In panel C, the experiment in (A) was quantitated with the levels of HIF-1α normalized to β-tubulin. Analysis of the data in panel C (n = 3 per group) revealed significant increases in HIF-1α following treatment with insulin vs. untreated control (p = 0.004, unpaired t-test) or XXI treated vs. insulin + XXI (p = 0.01). There was no difference between insulin treated vs. insulin plus XXI treated cultures (p = 0.29) although XXI treatment alone modestly decreased HIF-1α levels vs. untreated control (p = 0.006). Representative experiments are shown. Asterisks (*) indicate values that are significantly different from corresponding samples that were not treated with insulin.

Psen1 is best known for its role in γ-secretase activity [36]. Interestingly both the insulin [37] and IGF-1 [38] receptors are substrates for γ-secretase cleavage. In both cases these cleavages have been suggested to generate intracellular domains (ICDs) that may be involved in cellular signaling. To determine whether insulin induced activation of HIF-1α might require Psen1's associated γ-secretase activity, *Psen1+/+ *fibroblasts were treated with the γ-secretase inhibitor XXI for 16 hours in serum free media and then stimulated with insulin for four hours. As shown in Figure 5 (5A and 5C) HIF-1α decreased modestly following treatment with inhibitor XXI alone (p = 0.006, unpaired t-test) but was activated to the same extent whether inhibitor was present or not (p = 0.29). To ensure the adequacy of γ-secretase treatment blots were stripped and reprobed with an antibody that detects the C-terminal fragment (CTF) of N-cadherin. This fragment is generated by the sequential cleavage of N-cadherin by a metalloproteinase followed by γ-secretase and its appearance is stimulated when γ-secretase activity is blocked [39]. As expected, N-cadherin CTFs accumulated in *Psen1*+/+ samples treated with inhibitor XXI but not in untreated controls (Figure 5A, bottom panel). Cobalt chloride induced activation of HIF-1α was also not affected by treatment of *Psen1+/+ *fibroblasts with the γ-secretase inhibitor L-685,459 (Figure 5B). Thus impaired γ-secretase activity does not appear to explain the reduced HIF-1α induction in *Psen1-/- *cells.

### Normal activation of PI3K/Akt by IGF-1 and insulin in Psen1-/- fibroblasts

Growth factor stimulation principally increases HIF-1α protein synthesis by stimulating transcription/translation while degradation proceeds at a constant rate. The phosphatidylinositol 3-kinase (PI3K/Akt) pathway has been implicated as influencing HIF-1α induction [21,34]. Many of insulin and IGF-1's cellular effects are mediated by the PI3K/Akt pathway [40] and induction of HIF-1α by IGF-1 has been shown to be dependent on both PI3K/Akt and MAP kinase pathways [21,34]. In at least some cells, insulin's induction of HIF-1α also occurs through a PI3K/Akt dependent mechanism [19]. Psen1 is known to influence signaling through the PI3K/Akt pathway an effect that is γ-secretase independent [27,29]. Thus failed stimulation through the PI3K/Akt pathway might explain the lack of HIF-1α elevation in response to growth factors and if it affected constitutive HIF-1α synthesis might also explain the hyporesponsiveness of *Psen1-/- *fibroblasts to cobalt chloride if basal levels of HIF-1α synthesis are decreased.

**Figure 6:**
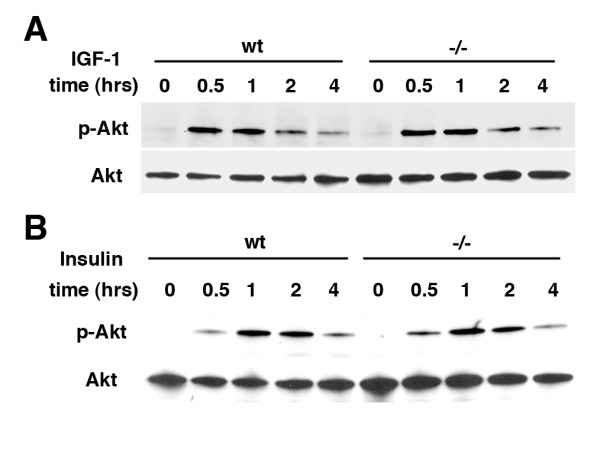
**Normal activation by IGF-1 and insulin of PI3K/Akt in *Psen1-/- *fibroblasts**. Immortalized fibroblast cell lines were treated with IGF-1 (A) or insulin (B) for the indicated times. Western blotting was performed for p-Akt (Ser473) followed by reprobing for total Akt. Panels show representative blots from experiments that were performed multiple times.

Therefore we determined whether PI3K/Akt signaling is impaired in response to IGF-1 or insulin in *Psen1-/- *fibroblasts. Interestingly as shown in Figure 6, both IGF-1 (A) and insulin (B) induced Akt phosphorylation at Ser473 on a similar time course and to a similar level in *Psen1+/+ *and *Psen1-/- *fibroblasts while total Akt levels remained unchanged. These studies thus suggest that Psen1's effects on HIF-1α induction are not based on defective signaling through PI3K/Akt pathway.

### Lower basal levels of HIF-1α in cells lacking Psen1

**Figure 7:**
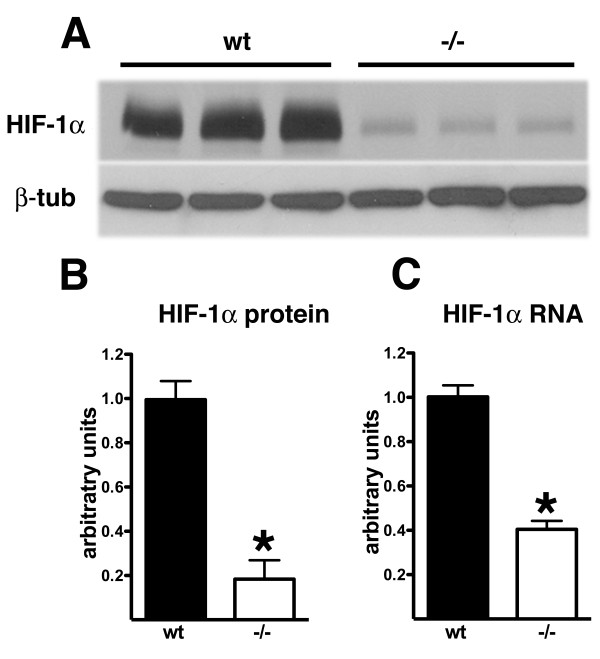
**Basal levels of HIF-1α are decreased in *Psen1-/- *fibroblasts**. Shown are prolonged exposures of Western blots from non-stimulated *Psen1+/+ *and *Psen1-/- *fibroblasts. A representative experiment that was performed multiple times is shown in panel A. In panel B, the experiment in (A) is quantified (asterisk indicates p = 0.0005, unpaired t-test). In panel C, levels of HIF-1α RNA were determined in *Psen1 +/+ *and *Psen1-/- *fibroblasts by qPCR (n = 3 cultures per group). Samples were run in triplicate and normalized to the geometric mean of Ppia and Gusb. Asterisk (*) indicates p = 0.0008.

If basal levels of HIF-1α were lower in *Psen1-/- *cells, this might affect the ability of cells to elevate HIF-1α protein in response to hypoxia as well as growth factor stimulation. To determine whether basal levels of HIF-1α protein were decreased in fibroblasts lacking *Psen1 *we examined prolonged exposures of Western blots from non-stimulated *Psen1+/+ *and *Psen1-/- *fibroblasts. As shown in Figure 7A and 7B, basal levels of HIF-1α protein were decreased by more than five-fold in *Psen1-/- *fibroblasts (p = 0.0005, unpaired t-test). We next determined HIF-1α RNA levels in *Psen1+/+ *and *Psen1-/- *immortalized fibroblasts by qPCR. As shown Figure 7C, levels of HIF-1α RNA were decreased by over 50% in *Psen1-/- *fibroblasts (p = 0.0008). Thus altered HIF-1α RNA levels might contribute to the decreased responsiveness of fibroblasts lacking Psen1.

### Accelerated degradation of HIF-1α in cells lacking Psen1

HIF-1α protein levels are determined by a balance between synthesis and degradation. Under normoxic conditions HIF-1α RNA is transcribed and translated at a constant rate producing a consistent amount of HIF-1α protein. However in normoxic conditions post-translational modifications by the prolyl hydroxylases (PHDs) induce ubiquitination and degradation of HIF-1α through the proteasome at a rate that exceeds the rate of synthesis [17]. Indeed under normoxic conditions HIF-1α has a very short half-life with little HIF-1α protein accumulating in normal cells [41].

**Figure 8:**
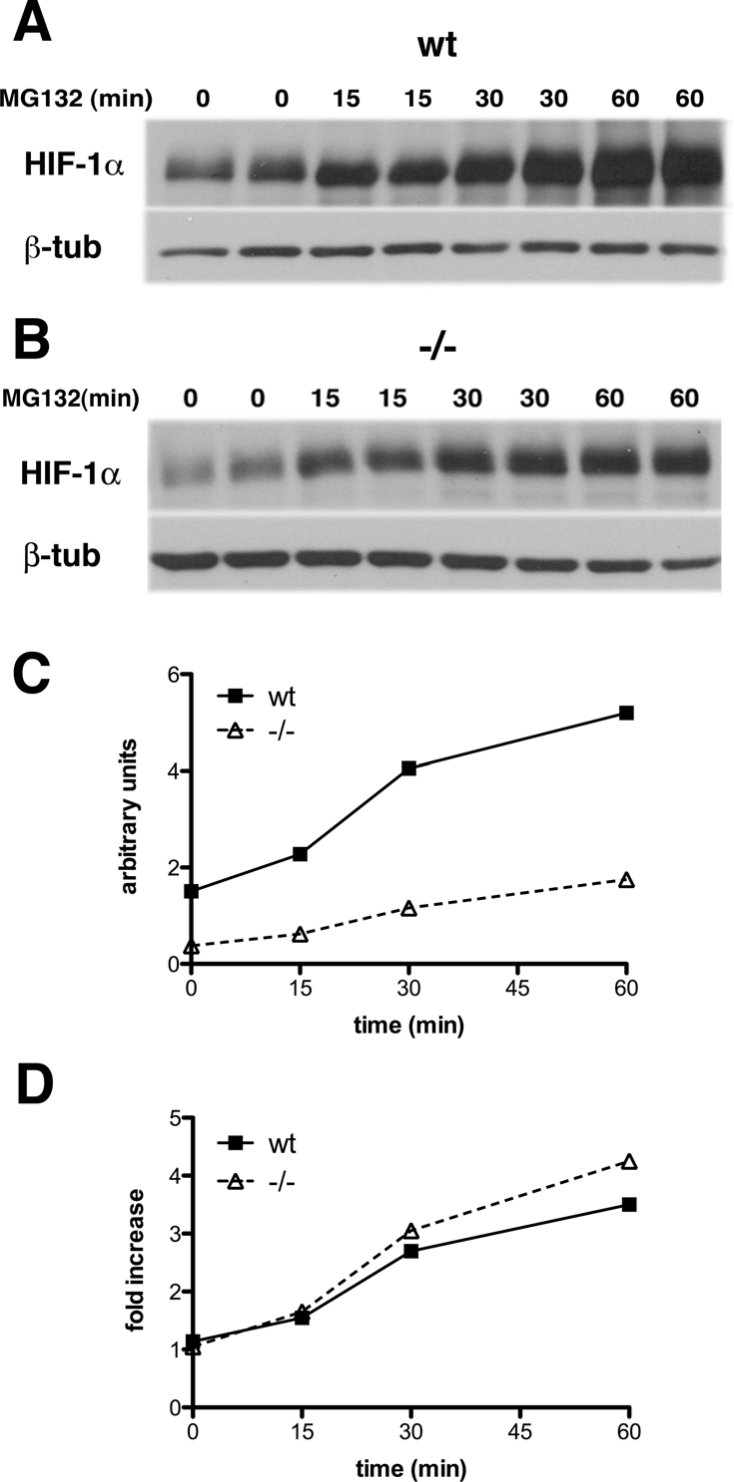
**Synthesis rates of HIF-1α are unchanged in *Psen1-/- *fibroblasts**. *Psen1+/+ *(A) and *Psen1-/- *(B) fibroblasts were treated with the proteasome inhibitor MG132 for the indicated times (min). Lysates were prepared and Western blotting was performed using an anti-HIF-1α antibody. The lower panels show the blots reprobed for β-tubulin. In panels C and D, the rate of accumulation of HIF-1α normalized to β-tubulin levels from the experiments in panels A and B is shown either as the raw data (C) or normalized to basal levels of HIF-1α in the respective cell types (D). Note that although basal levels of HIF-1α are lower in *Psen1-/- *fibroblasts, HIF-1α accumulated at similar rates in *Psen1+/+ *and *Psen1-/- *fibroblasts. A representative example is shown from experiments that were performed multiple times.

If the low levels of HIF-1α in *Psen1-/- *fibroblasts were due solely to decreased HIF-1α synthesis as might be suggested by the lowered HIF-1α RNA levels then HIF-1α protein should accumulate more slowly in *Psen1-/- *fibroblasts if protein degradation was blocked. We tested this hypothesis by blocking proteasomal degradation of HIF-1α in *Psen1+/+ *and *Psen1-/- *fibroblasts with the proteasome inhibitor MG132. However, as shown in Figure 8, although starting at lower basal levels, HIF-1α protein accumulated at approximately the same rate in *Psen1+/+ *and *Psen1-/- *fibroblasts.

**Figure 9:**
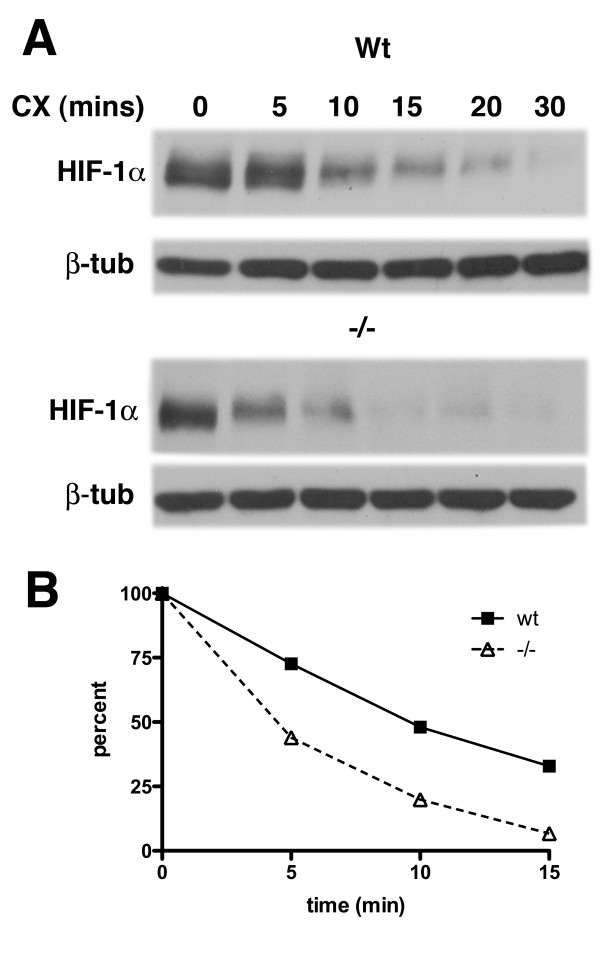
**Accelerated degradation of HIF-1α in *Psen1-/- *fibroblasts**. *Psen1+/+ *and *Psen1-/- *fibroblasts were treated with cycloheximide. In panel A, a representative experiment is shown with cells treated for the indicated times. Due to the difference in basal HIF-1α levels between *Psen1 +/+ *and *Psen1-/- *cells, total protein loaded (15 μg for *Psen1+/+ *and 30 μg for *Psen1 -/-*) and blot exposure times were adjusted so that the signal at time 0' would be of similar intensity in *Psen1+/+ *and *Psen1-/- *cells to facilitate comparison. In panel B, the % HIF-1α remaining at each time point normalized to β-tubulin is shown. Data is derived from the average of three independently performed experiments.

This finding raised the possibility that Psen1 might influence basal HIF-1α levels not through effects on synthesis but rather by affecting HIF-1α degradation. We initially attempted measuring HIF-1α half-life in *Psen1+/+ *and *Psen1-/- *fibroblasts by pulse-chase experiments using ^35^S methionine/cysteine followed by immunoprecipitation (IP) of HIF-1α. However we could not detect HIF-1α in either *Psen1+/+ *or *Psen1-/- *fibroblasts (data not shown), an effect that we suspect is due to the low basal levels of HIF-1α in most cells. Therefore to determine whether HIF-1α stability was affected by the absence of Psen1, we treated *Psen1+/+ *and *Psen1-/- *fibroblasts with cycloheximide to block HIF-1α translation and measured the rate of HIF-1α degradation. As shown in Figure 9, the rate of HIF-1α degradation was higher in *Psen1-/- *fibroblasts leading to a decrease in HIF-1α half-life from ≈10 min in *Psen1+/+ *fibroblasts to less than 5 min in *Psen1-/- *fibroblasts. Thus degradation of HIF-1α protein is accelerated in cells that lack Psen1.

### Physical interaction between Psen1 and HIF-1α

Psen1 is known to interact with a number of proteins [42] with this interaction in some cases protecting proteins from degradation through the proteasome [43] suggesting that one possible explanation for the effects of Psen1 on HIF-1α stability might be that Psen1 and HIF-1α physically interact and that this interaction protects HIF-1α from proteasomal degradation. We therefore explored whether Psen1 and HIF-1α might interact by co-transfecting Psen1 and HIF-1α cDNAs into HEK 293 cells.

**Figure 10:**
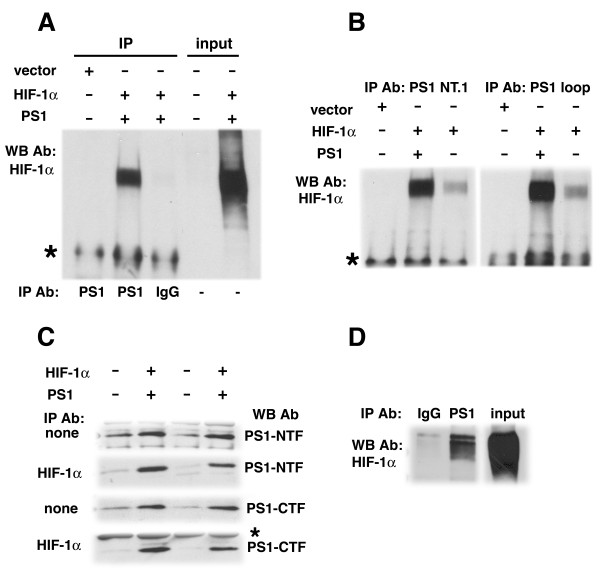
**Physical interaction between HIF-1α and Psen1**. Expression vectors carrying no insert, Psen1 or HIF-1α were transfected into HEK 293 cells in the indicated combinations. In panel A, immunoprecipitations (IP) were performed with a polyclonal anti-Psen1 antibody which recognizes the Psen1 NTF or with rabbit IgG as control and Western blotting was performed for HIF-1α. Total extracts without IP (-) (5% of input) were loaded in the two far right lanes (input). Note the co-immunoprecipitation of HIF-1α in the cultures co-transfected with HIF-1α and Psen1. A similar experiment was performed in (B), except that immunoprecipitations were performed with the monoclonal antibody NT.1 which recognizes the Psen1 NTF or with an antibody against the Psen1 loop region. In panel C, immunoprecipitation was done with a monoclonal HIF-1α antibody followed by Western blotting with an antibody against the Psen1 NTF (NT.1) or CTF (33B10). Panels labeled "none" show blots of total extracts prior to immunoprecipitation (input). Bands marked with an asterisk (*) are likely IgGs based on the rate of migration. Panel D shows the co-immunoprecipitation of endogenous HIF-1α by endogenous Psen1. Wild type fibroblasts were treated for 4 hours with 100 μM CoCl_2 _and 10 μM MG132 to induce HIF-1α accumulation and co-immunoprecipitations were performed as in (A).

As shown in Figure 10, when co-transfected cultures were immunoprecipitated with Psen1 antibodies that recognize either the Psen1 NTF or the loop domain, Western blotting showed co-immunoprecipitation of HIF-1α (Figure 10A, B). In the reciprocal experiment (Figure 10C), immunoprecipitation of HIF-1α, followed by Western blotting for Psen1 showed co-immunoprecipitation of Psen1. In addition, as shown in Figure 10B endogenous Psen1 co-immunoprecipitated a transfected HIF-1α (last lane to the right in each panel). Immunoprecipitation of endogenous HIF-1α with anti-Psen1 antibodies could also be detected in fibroblasts pretreated with CoCl_2 _and MG132 (Figure 10D). Thus these experiments show that Psen1 and HIF-1α can interact, providing support for the notion that Psen1 may affect HIF-1α stability by protecting it from degradation through a direct physical interaction.

### Impaired activation of HIF1-α in fibroblasts harboring the M146V Psen1 FAD mutation

*Psen1 *associated FAD mutations are generally thought of as gain of function mutations that exert their deleterious effect by increasing production of the longer more amyloidogenic Aβ species [1]. However studies have suggested that in other contexts *Psen1 *FAD mutations may actually be hypofunctional and indeed with regard to γ-secretase function, *Psen1 *FAD mutations are generally less efficient than wild type *Psen1 *in promoting γ-secretase activity [44,45].

Since FAD mutant effects on induction of HIF-1α could have pathophysiological consequences in AD, we determined whether *Psen1 *associated FAD mutants might affect HIF-1α induction using immortalized fibroblasts that express either human wild type Psen1 or the M146V FAD mutant. These lines were generated from transgenic mice that express either wild type human Psen1 from a P1 artificial chromosome (PAC) or FAD mutant Psen1 generated by retrofitting the PAC with the M146V FAD mutation [46]. To eliminate the effects of endogenous mouse Psen1, both PAC transgenes were bred onto the mouse *Psen1-/- *background and fibroblast cell lines were established from transgenic mice. These transgenic lines express equivalent amounts of wild type and M146V FAD mutant proteins as judged by Western blotting [46].

**Figure 11:**
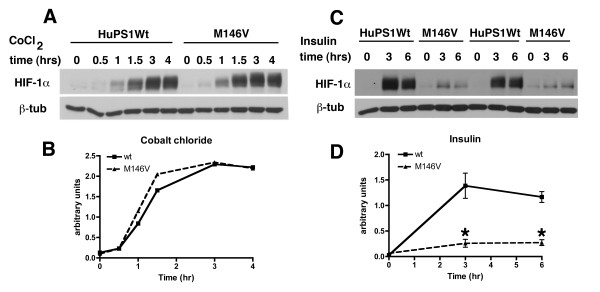
**Altered activation of HIF-1α in fibroblasts harboring the M146V PSEN1 FAD mutation**. Fibroblast cell lines harboring human wild type Psen1 (HuPS1Wt) or the M146V FAD mutant both on the mouse *Psen1-/- *background were treated with cobalt chloride (A) or insulin (C) as in Figure 1 for the indicated times (hours). Representative blots are shown from experiments that were performed multiple times. The experiment in panel A is quantitated in panel B. In panel D, the insulin induction time course in panel C was quantitated. Asterisks (*) indicate p = 0.04 at 3 hours and p = 0.02 at 6 hours (unpaired t-tests).

An equally robust activation of HIF-1α was seen in both wild type and M146V FAD mutant fibroblasts in response to cobalt chloride (Figure 11A and 11B). By contrast, when cultures were treated with insulin, fibroblasts harboring the M146V FAD mutation were hyporesponsive. While HIF-1α increased more than 10-fold in fibroblasts with the wild type human Psen1, there was minimal induction of HIF-1α in the M146V FAD mutant cells (Figure 11C and 11D). Thus hypoxic induction of HIF-1α is intact in fibroblasts harboring a *Psen1 *FAD mutant while metabolic induction is defective.

**Figure 12:**
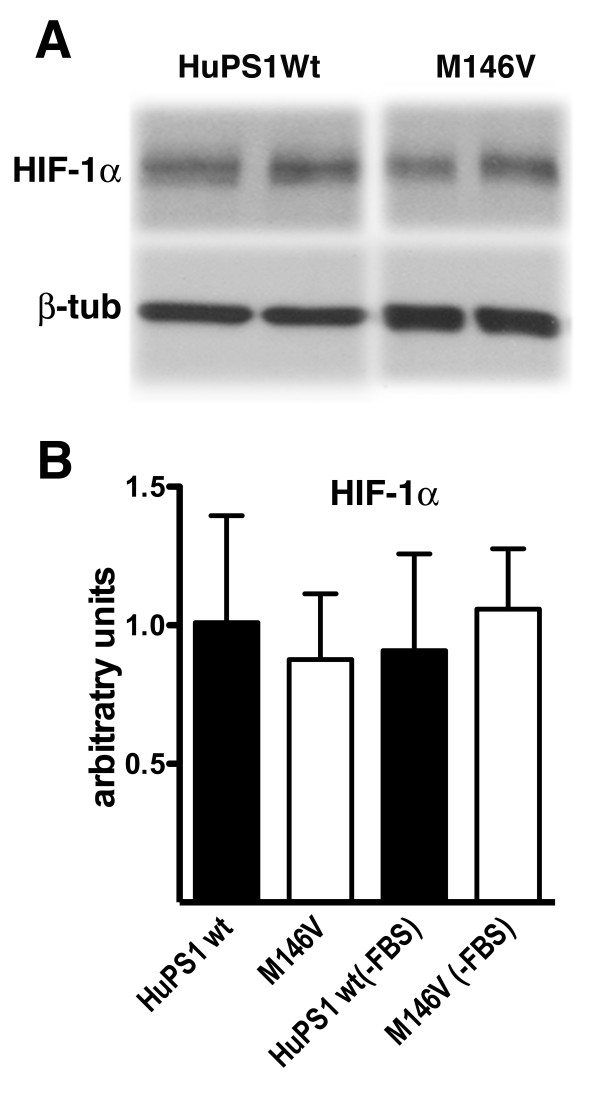
**Basal levels of HIF-1α are unchanged in M146V FAD mutant fibroblasts**. Shown are prolonged exposures of Western blots from the fibroblast lines studied in Figure 11. In panel A, representative blots are shown of samples from individual cultures harboring the human wild type Psen1 (HuPS1Wt) or the M146V FAD mutant. The lower panel shows the blot reprobed for β-tubulin. In panel B, levels of HIF-1α were determined by quantitative Western blotting (n = 5 cultures per genotype) with the levels of HIF-1α normalized to the levels of β-tubulin. Studies were performed both in the presence of serum (FBS) and after overnight serum starvation (-FBS). There were no statistically significant differences between the HuPS1Wt and M146V FAD mutant cell lines.

To determine whether basal levels of HIF-1α protein were altered in M146V FAD fibroblasts we examined prolonged exposures of Western blots from fibroblasts expressing wild type human Psen1 and the M146V FAD mutant. As shown in Figure 12, levels of HIF-1α were not significantly different between fibroblasts expressing human wild type and M146V FAD mutant Psen1 (p = 0.72). These observations thus argue that the effects of FAD mutant Psen1 on HIF-1α are distinct from the effects of null mutations in Psen1 and that while absence of Psen1 reduces basal HIF-1α synthesis, in FAD mutant cells basal synthesis is intact but metabolic induction is impaired.

### Altered insulin-receptor activation in cells harboring the Psen1 FAD mutant M146V

**Figure 13:**
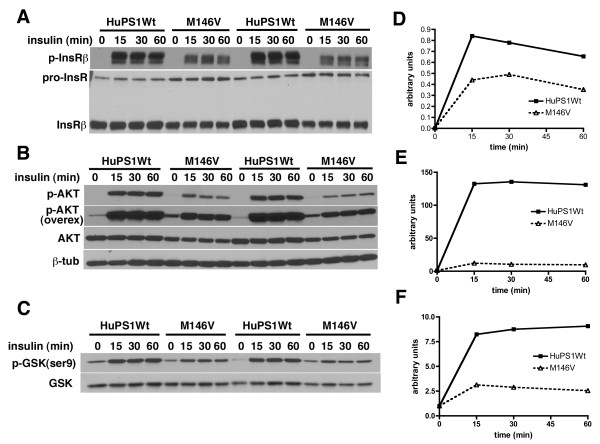
**Decreased insulin-induced activation of the insulin receptor, Akt and GSK-3β in fibroblasts harboring the M146V Psen1 FAD mutation**. In panel A, human wild type Psen1 (HuPS1Wt) or M146V FAD mutant cell lines were serum starved overnight and then stimulated with insulin for the indicated times. Western blotting for the phosphorylated IR receptor β (Tyr 1150/1151) or total IR is shown in experiments run in duplicate. The pro-insulin receptor and processed IR are indicated. In panel B, duplicate experiments are shown following Western blotting for p-Akt (Ser473) and total Akt. p-Akt panels are shown at two different exposures. Lower panel shows the blot reprobed for β-tubulin as a loading control. In panel C, the extracts were blotted and probed for p-GSK-3β (Ser9) and total GSK-3β. In panels D, E and F the relative increase in p-IR/total IR (D), p-Akt/total Akt (E) and p-GSK-3β/total GSK-3β (F) are shown for the experiments in A-C.

We next determined whether lack of HIF-1α induction in M146V FAD mutant fibroblasts might be related to impaired signal transduction through the insulin receptor (IR). Binding of insulin to the α subunit of the IR causes a conformational change in the receptor that induces autophosphorylation of tyrosine residues in the β subunit. Figure 13A shows duplicate experiments in which fibroblasts expressing human wild type and M146V FAD mutant Psen1 were treated with insulin and demonstrates that following treatment IR-β is less phosphorylated in M146V fibroblasts suggesting decreased receptor activation. Since many of the signaling effects of insulin are mediated through insulin's activation of the protein kinase B/Akt pathway [40] we assessed the phosphorylation of Akt and its target glycogen synthase kinase-3β (GSK-3β). As shown in Figure 13B, Akt was hypophosphorylated following insulin stimulation of M146V FAD mutant containing cells. A similar result was also seen for GSK-3β (Figure 13C). Thus responsiveness of the insulin signal transduction pathway is generally dampened in fibroblasts harboring the M146V FAD mutation, offering a potential explanation for the impaired activation of HIF-1α.

### Psen1-/- primary neuronal cultures activate HIF-1α normally in response to cobalt chloride but are defective in HIF-1α induction by insulin and IGF-1

**Figure 14:**
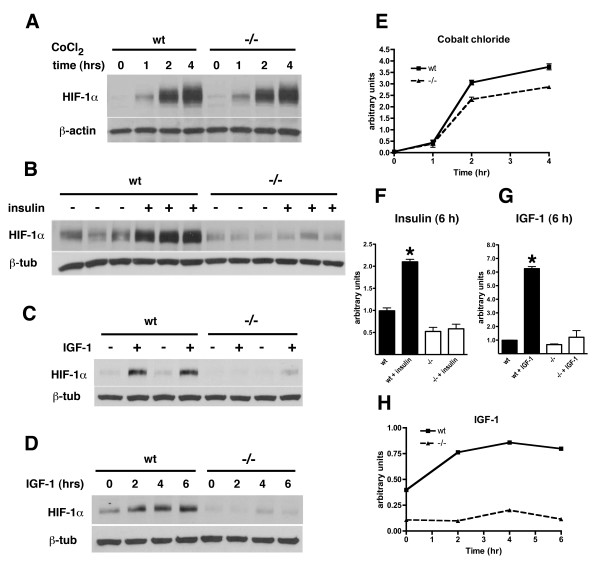
**Altered induction of HIF-1α in *Psen1-/- *primary neuronal cultures**. Primary neuronal cultures were prepared from E15.5-16 embryonic brains and maintained in Neurobasal medium/B27 supplement. Experiments were performed after 5-6 days *in vitro*. In panel A, cultures were treated with 100 μm cobalt chloride for the indicated times (hrs). In panels B and C, cells were cultured without B27 overnight and then treated with insulin (B) or IGF-1 (C) for six hours. In panel D, cells were cultured without B27 overnight and then treated with IGF-1 for the indicated times. Western blotting for HIF-1α was performed as in Figure 1 and the lower panels show the same blots reprobed for β-tubulin or actin. In panels E and H, the time course of the studies in (A) and (D) are quantitated. The insulin and IGF-1 studies in panels B and C are quantitated in (F) and (G). All panels show representative blots from experiments that were performed multiple times. Asterisks indicate p < 0.05 vs. untreated control (unpaired t-tests).

To determine whether HIF-1α induction was impaired in those cells most relevant to AD, namely neuronal cells, we prepared primary neuronal cultures from wild type and *Psen1-/- *embryos and at 5-6 days *in vitro *treated them with cobalt chloride, insulin or IGF-1. As shown in Figure 14A and 14E, unlike fibroblasts, an equally robust activation of HIF-1α was seen in both wild type and *Psen1-/- *neuronal cultures in response to cobalt chloride. By contrast in experiments in which cultures were treated with insulin (Figure 14B, F) or IGF-1 (Figure 14C, D, G, H), *Psen1-/- *neuronal cultures were hyporesponsive. While HIF-1α increased more than two-fold in wild type neurons in response to insulin (p = 0.003) and six-fold in response to IGF-1 (p = 0.01), there was no significant change in HIF-1α following stimulation of *Psen1-/- *neurons (p = 0.76 insulin; p = 0.76 IGF-1).

**Figure 15:**
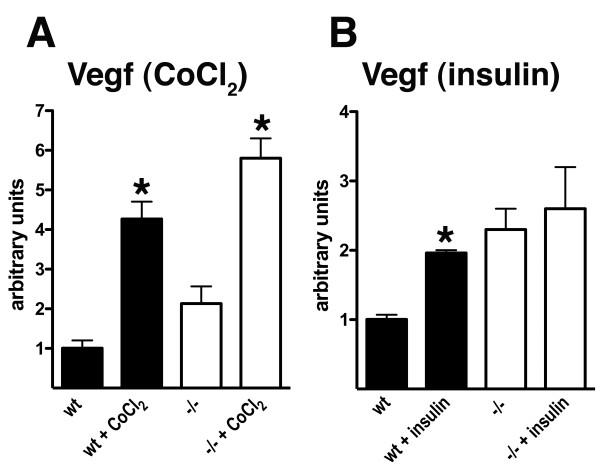
**Activation of HIF-1α downstream target genes in *Psen1-/- *neurons treated with cobalt chloride or insulin**. Levels of Vegf RNA were determined by qPCR following three hours of treatment of wild type or *Psen1-/- *primary neuronal cultures with cobalt chloride (A, n = 3 cultures per group) or seven hours with insulin (B, n = 2 cultures per group). Samples were run in triplicate and normalized to Ppia. Asterisks indicate p < 0.01 vs. untreated control (unpaired t-tests).

Consistent with the Western blotting data, Vegf RNA was induced in *Psen1-/- *neurons as in *Psen1+/+ *at 3 hours after cobalt chloride stimulation (p = 0.007, Figure 15A). Interestingly Vegf RNA tended to increase in *Psen1-/- *neurons compared to *Psen1+/+ *following the overnight removal of B27 before insulin treatment (p = 0.06, Figure 15B) but did not increase further in *Psen1-/- *neurons following insulin stimulation (p = 0.75) in contrast to the approximately two-fold increase seen in *Psen1+/+ *neurons (p = 0.007). Thus unlike fibroblasts, in neuronal cells hypoxic induction of HIF-1α is intact while as in fibroblasts metabolic induction is defective.

**Figure 16:**
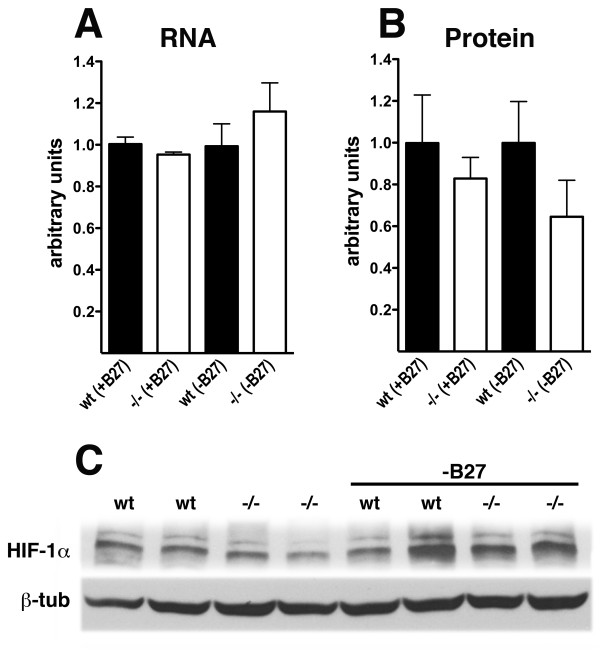
**Basal levels of HIF-1α RNA and protein are not significantly changed in *Psen1-/- *neurons**. Basal HIF-1α RNA and protein were measured in neurons in the presence and absence of B27 supplement. In panel A, HIF-1α RNA was measured in neuronal cultures (n = 3/genotype) by qPCR. Samples were run in triplicate and normalized to the geometric means of Ppia and Gusb. In panel B, basal levels of HIF-1α protein were measured in neurons by Western blotting (n = 6 for cultures with B27 and n = 10 for B27 starved cultures). No statistically significant differences were found among the samples (unpaired t-tests). In panel C, representative Western blots are shown of cells cultured with B27 or without (-B27).

**Figure 17:**
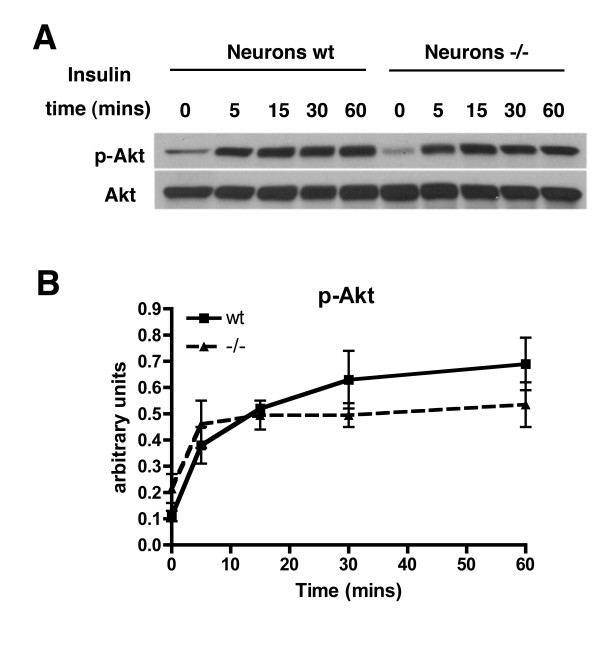
**Normal activation by insulin of PI3K/Akt in *Psen1-/- *neurons**. Primary neuronal cultures were treated with insulin for the indicated times. In panel A, Western blotting was performed for p-Akt (Ser473) followed by reprobing for total Akt. Shown are representative blots from experiments that were performed multiple times. In panel B, the time course of induction of p-Akt in primary neuronal cultures (n = 2 per genotype per time point) is shown as a ratio of p-Akt/total Akt. There were no significant differences at any time point (unpaired t-tests).

Also unlike fibroblasts, basal levels of HIF-1α RNA and protein were not significantly different between *Psen1+/+ *and *Psen1-/- *neurons (Figure 16A, B) and were not affected by the removal of B27. As in fibroblasts no significant difference was seen between *Psen1+/+ *and *Psen1-/- *neurons in the phosphorylation of Akt following insulin stimulation (Figure 17).

### HIF-1α is activated normally during development in Psen1-/- embryos

**Figure 18:**
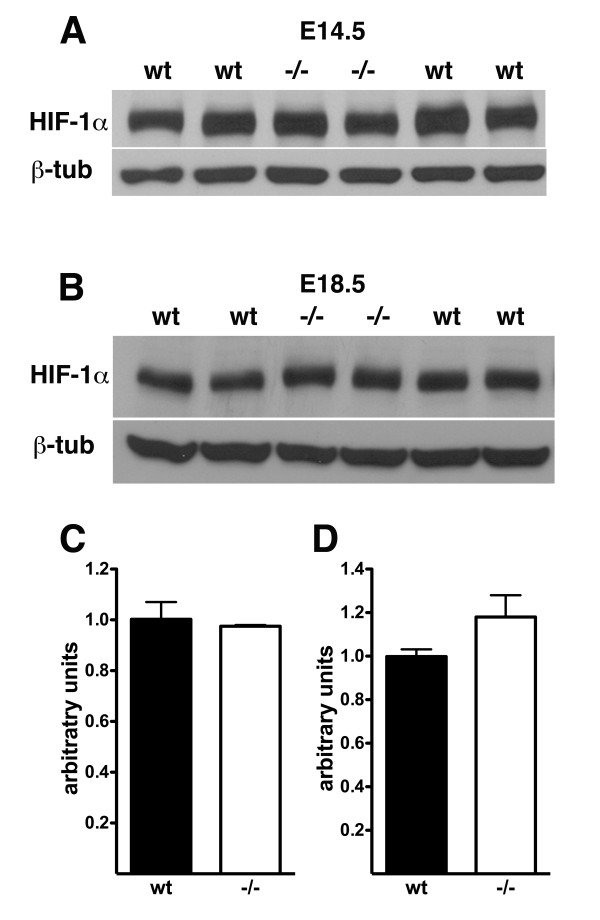
**No change in HIF-1α levels in *Psen1-/- *embryonic brain**. HIF-1α levels were determined by Western blotting of extracts from E14.5 (A) or E18.5 (B) brain from wild type (wt) or homozygous (-/-) *Psen1 *mutant mice. Representative experiments are shown. Lower panels show the same blots reprobed for β-tubulin. HIF-1α levels were quantitated as a ratio to β-tubulin at E14.5 (C) and E18.5 (D) (n = 4 wild type and 2 *Psen1*-/- at each age). There were no differences between wild type (wt) and *Psen1-/- *at either age (unpaired t-tests).

Hypoxia is regarded as a strong physiological activator of HIF-1α during normal development including in the CNS [47]. To determine whether HIF-1α was activated *in vivo *in the absence of Psen1 we performed Western blotting on wild type and *Psen1-/- *embryos. Extracts from whole brain at E14.5 and E18.5 were examined (Figure 18). HIF-1α expression was easily detectable at both ages in wild type as well as *Psen1-/- *embryos and levels did not significantly vary between genotypes (p = 0.71, E14.5; p = 0.08, E18.5). Thus there appears to be no defect to HIF-1α activation due to the lack of *Psen1 in vivo*. However since *in vivo *activation at this age is likely driven primarily by hypoxia, these findings can be seen as consistent with the preservation of hypoxic induction of HIF-1α in *Psen1-/- *neuronal cultures *in vitro*.

## Discussion

Psen1 is a polytopic transmembrane protein that was first discovered because of its association with FAD [2,48]. Psen1 influences multiple molecular pathways being best known for its role as a component of the γ-secretase complex [48]. HIF-1 is a transcription factor that was first recognized as a DNA-binding protein that mediates hypoxia-inducible expression of the erythropoietin (EPO) 3' enhancer. It is now known that HIF-1 is a key transcriptional regulator of a wide variety of cellular genes that are induced by and regulate cellular responses to hypoxia [16,17,49,50].

Here we show that Psen1 regulates induction of HIF-1α. Specifically we demonstrate that there is impaired induction of HIF-1α in fibroblasts lacking Psen1 following stimulation with the hypoxia mimetic cobalt chloride as well as treatment with insulin and calcium chelation. Associated with impaired induction there was reduced and delayed activation of the HIF-1α target genes Vegf and Glut-1. We further show that decreased basal levels of HIF-1α best account for this impairment in fibroblasts lacking Psen1.

Depressed levels of basal HIF-1α in *Psen1-/- *fibroblasts may in part be the result of lowered levels of HIF-1α RNA. However the ≈ 50% reduction in HIF-1α RNA levels did not appear sufficient to explain the more than 5-fold reduction in basal levels of HIF-1α protein in *Psen1-/- *fibroblasts. Rather these results suggested that in addition either constitutive synthesis of HIF-1α was decreased at a post-transcriptional level or that constitutive degradation was increased. In further studies we found that increased degradation best accounts for these effects showing that when proteasomal degradation of HIF-1α was blocked, HIF-1α levels accumulated as rapidly in *Psen1-/- *fibroblasts as in *Psen1+/+ *cells. By contrast when translation was blocked with cycloheximide levels of HIF-1α decreased more rapidly in *Psen1-/- *fibroblasts than *Psen1+/+ *with an approximate 50% reduction in HIF-1α half-life.

Mechanistically we show that Psen1's γ-secretase activity is not needed for HIF-1α induction by insulin nor cobalt chloride and that in the case of insulin induction of HIF-1α, impaired signaling through the PI3K/Akt pathway cannot account for the effect. Rather we show that Psen1 and HIF-1α physically interact suggesting that Psen1 may stabilize HIF-1α and protect it from degradation.

Both mouse and human Psen1 are initially produced as an ≈ 45-48 kDa holoprotein. However, in most cells and tissues nearly all of the holoprotein is cleaved into an ≈ 30 kDa NTF and an ≈18 kDa CTF, that are produced by endoproteolytic cleavage within a large cytoplasmic loop region [48]. NTFs and CTFs associate with each other as heterodimeric components of a larger multimeric protein complex. After incorporation into these high molecular weight complexes, NTFs and CTFs remain associated and attain relatively long half-lives.

Our co-IP studies suggest that HIF-1α associates with both the Psen1 NTF and CTF and likely forms a part of these complexes. Psen1 holoprotein that remains monomeric is rapidly degraded by a proteasome-dependent mechanism [51,52] and complex incorporation is likely necessary for the biological activity of presenilins [48]. Psen1 and 2 proteolysis is also regulated by the proteasome interacting proteins ubiquilin-1 and -2 [53-55] and Psen1 influences proteasomal degradation of another component of the γ-secretase complex Pen-2 [43]. Indeed the effects of Psen1 on Pen-2 appear relatively similar to the effects of Psen1 on HIF-1α with in both cases the absence of Psen1 leading to accelerated protein degradation through the proteasome. Interestingly, overexpression of Psen1 has also been reported to increase levels of HIF-1β in pancreatic islet cells [56] pointing to a possibly larger role of Psen1 in HIF-1 signaling although the mechanistic basis for Psen1's effect on HIF-1β is unknown.

In contrast to the more global effects that the absence of Psen1 has in fibroblasts, hypoxic signaling proceeded normally in *Psen1-/- *neurons and in fibroblasts harboring the M146V FAD mutation while in these same cells metabolic signaling through insulin and IGF-1 was impaired. HIF-1α levels were also normal in *Psen1-/- *embryos arguing that activation in neuronal cells during development which is probably mostly driven by hypoxia [47] can proceed normally without Psen1.

While hypoxia regulates HIF-1α levels mostly through effects on protein degradation by the proteasome, metabolic/growth factor stimulation principally increases HIF-1α protein synthesis by stimulating transcription/translation while degradation proceeds at a constant rate [33]. Of these two pathways, hypoxic induction of HIF-1α results in more dramatic elevations of HIF-1α than metabolic/growth factor stimulation likely because degradation is the key rate limiting step in setting basal HIF-1α levels while factors such as insulin and IGF-1 induce HIF-1α by stimulating synthesis on a background of continued degradation.

Interestingly, unlike in *Psen1-/- *fibroblasts, basal levels of HIF-1α were not reduced in fibroblasts harboring the *M146V *FAD mutation or in *Psen1-/- *primary neurons. While the basis for these differences in HIF-1α basal levels remains to be explained, they provide a potential explanation for why HIF-1α responsiveness differs between cell types in that hypoxic induction of HIF-1α depends on a basal level of HIF-1α being present. Metabolic/growth factor induction being weaker requires a higher basal level of HIF-1α to be present in order to produce detectible induction. Since *Psen1-/- *fibroblasts have dramatically reduced levels of basal HIF-1α neither hypoxic nor metabolic/growth factor induction is possible. By contrast in FAD mutant fibroblasts or *Psen1-/- *primary neurons, a basal level of HIF-1α is present that is sufficient to support hypoxic induction while metabolic/growth factor induction is impaired.

Of the metabolic pathways implicated in HIF-1α regulation the PI3K/Akt and MAP kinase are the best-documented [33] and many of insulin's as well as IGF-1's cellular effects are mediated by the PI3K/Akt pathway [40]. In at least some cells, insulin's induction of HIF-1α has been shown to occur through a PI3K/Akt dependent mechanism [19] and induction of HIF-1α by IGF-1 has also been shown to be dependent on PI3K/Akt activation [21,34]. Psen1 is known to influence signaling through the PI3K/Akt pathway, an effect that is γ-secretase independent [27,29].

Interestingly we did not find effects on PI3K/Akt induction in *Psen1-/- *fibroblasts or neurons although we found that in fibroblasts harboring the *Psen1 M146V *FAD mutant insulin receptor activation as well as activation of PI3K/Akt and Akt's downstream target GSK-3β were impaired. Thus while a direct effect of Psen1 on insulin/IGF-1 signaling might explain the failed activation of HIF-1α in FAD mutant fibroblasts, it cannot account for the failed activation in *Psen1-/- *neurons. Future studies will be needed to sort out the cell type specific differences as well as the differences between the effects of the absence of Psen1 and the presence of Psen1 FAD mutants.

The implications of these observations for the pathophysiological effects of *Psen1 *FAD mutations remains speculative. Insulin receptors are expressed widely in brain including areas involved in AD such as the hippocampus [57]. The widespread expression of insulin receptors in brain suggests that insulin signaling plays a significant functional role in brain and indeed signaling through the insulin receptor is known to influence a wide variety of cellular functions related to cellular homeostasis and growth factor signaling including affecting synaptic transmission and neurotransmitter levels as well as exerting effects on learning and memory [58,59]. IGF-1 signaling is also of interest since it has many links to brain function as well as potential pathophysiological connections to neurodegenerative diseases including AD [60-63]. There has also been much interest in the potential role of insulin resistance in AD with most epidemiologic studies suggesting diabetes to be a risk factor for AD [64]. Diabetes and anti-diabetic medication have also been shown to influence AD related neuropathology [65,66].

Both insulin and IGF-1 signaling have connections to Aβ production [67-74] and tau phosphorylation [75-80] as well as have roles in modulating neural inflammatory reactions and neuronal apoptosis [81,82]. Thus an effect of Psen1 FAD mutations on insulin and IGF-1 related signaling could impact AD-related pathology including but not limited to effects on HIF-1α induction.

## Conclusions

Yet whatever the pathophysiological implications for Psen1 associated FAD mutations, our observations show that Psen1 is needed for the normal induction of HIF-1α and that at least one Psen1 FAD mutation impairs aspects of HIF-1α induction. Future studies in Psen1 FAD mutant mice will be needed to further clarify the role that HIF-1α signaling may play in the pathophysiological changes seen in these mice [83].

## Materials and methods

### Genetically modified mice

The *Psen1-/- *mice utilized were those generated by Shen et al. [84]. These animals were provided on a mixed genetic background and have been maintained by breeding to C57BL/6 wild type mice. Genotypes were determined by PCR utilizing primers from the *Psen1 *intron 1 (5'ACCTCAGCTGTTTGTCCCGG3'), the neo gene (5'GCACGAGACTAGTGAGACGTG3') and *Psen1 *exon 3 (5'TCTGGAAGTAGGACAAAGGTG3') as described in Shen et al. [84]. Heterozygous mice were mated to produce *Psen1-/- *embryos with the day a vaginal plug was detected designated as E0.5. Pregnant female mice were euthanized with carbon dioxide and *Psen1-/- *embryos were presumptively identified based on their gross dysmorphic appearance. A portion of the body was saved and used to isolate DNA and confirm genotypes. Due to the mixed genetic background, *Psen1+/+ *and *Psen1+/- *littermates were used as controls. Since *Psen1+/- *mice have never been observed to exhibit any developmental abnormalities, they were treated as wild type controls.

Transgenic mice expressing wild type human Psen1 from a P1 bacteriophage artificial chromosome (PAC) were produced using the clone RP1-54D12 (204 kb; accession number AC 006342) containing the entire human Psen1 transcription unit (> 75 kb). Founders were generated by injecting C57BL/6 × C3 H F_1 _oocytes. In order to generate PAC transgenic mice expressing the M146V FAD mutation, the 54D12 clone was retrofitted using the Rec A-mediated homologous recombination system described by Ali Imam et al. [85] and transgenic founders were generated as described above. Further details concerning production and characterization of the *Psen1 *wild type and FAD mutant PAC transgenic mice can be found elsewhere [46]. All protocols were approved by the Institutional Animal Care and Use Committees of the James J. Peters Department of Veterans Affairs Medical Center (Bronx, NY, USA) and the Mount Sinai School of Medicine (New York, NY, USA) and were conducted in conformance with Public Health Service (PHS) policy on the humane care and use of laboratory animals and the NIH Guide for the Care and Use of Laboratory Animals.

### Cell culture

Generation of immortalized *Psen1+/+ *and *Psen1-/- *mouse embryonic fibroblasts has been described previously [27]. Immortalized fibroblast lines were also developed from the Psen1 wild type and M146V PAC transgenic mice described above. In order to eliminate effects of the endogenous mouse *Psen1*, both PAC transgenic lines were bred onto the mouse *Psen1-/- *background and fibroblast cell lines were established from E16.5 embryos. After removing the head and liver, embryos were finely minced and treated with 0.25% trypsin (Invitrogen, Carlsbad, CA, USA), 0.1% EDTA. Trypsinized tissues were plated in Dulbecco's Modified Eagle's Medium (DMEM) supplemented with 20% heat-inactivated fetal calf serum (Mediatech, Manassas, VA, USA), penicillin, streptomycin, fungizone, and glutamine (Invitrogen) at 37°C in 5% CO_2_. Explanted cells were continuously passed in the same medium until lines were established. Fibroblast cell lines lacking both presenilins (*Psen-/-*) were a gift from Dr. Nikolaos Robakis (Mount Sinai School of Medicine, New York, NY, USA).

Immortalized fibroblasts were maintained in DMEM medium supplemented with 20% FCS at 37°C and 5% CO_2_. To study HIF-1α induction under hypoxic conditions confluent cells were treated with the hypoxia mimetic CoCl_2 _(100 μM, Sigma Aldrich, St. Louis, MO, USA) for 4 hrs. For insulin or IGF-1 treatment, cells were serum starved overnight and treated with 2.4 μg/ml of insulin (porcine pancreas, Sigma Aldrich) or 100 ng/ml IGF-1 (Sigma Aldrich) for 4-6 hours. Cells were washed with phosphate buffered saline (PBS) and lysed in 10 mM Na phosphate buffer, pH 7.4, 150 mM NaCl, 2 mM EDTA, 1%Triton X-100, 0.25% Na deoxycholate, and 0.5% SDS, supplemented with HALT protease inhibitor cocktail (Pierce, Rockford, IL, USA) and phosphatase inhibitor cocktails I and II (Sigma Aldrich). The lysates were briefly sonicated and centrifuged at 14,000 rpm for 15 min. The cleared supernatant was then collected. Protein concentrations were determined with the BCA reagent (Pierce).

In some experiments *Psen1 +/+ *and *Psen1-/- *fibroblasts at ≈70% confluence were treated with 5 μM 1,2-bis(o-aminophenoxy)ethane-N,N,N',N'-tetraacetic acid tetrakis (acetoxymethylester) (BAPTA-AM, Sigma Aldrich), 10 μm MG132 (Calbiochem, San Diego, CA, USA), or 100 μg/ml cycloheximide (Sigma-Aldrich). For γ-secretase inhibition experiments, *Psen1 +/+ *cultures were treated overnight with 1 μM inhibitor XXI (also known as compound E, Calbiochem) or 1 μM L-685,458 (Calbiochem).

Primary neuronal cultures were prepared from the cerebral cortex isolated from E15.5-16 embryos as previously described [86,87] and maintained in Neurobasal medium/B27 supplement (Invitrogen) for 5-7 days *in vitro *(DIV). For treatment with insulin or IGF-1, neurons were maintained overnight in neurobasal medium devoid of B27 supplement and then treated as described above.

### Tissue harvesting

Brains were homogenized in 10 volumes of RIPA buffer **(**50 mM Tris HCl, pH 7.6, 0.15 M NaCl, 1 mM EDTA, 1% Triton X100, 1% sodium deoxycholate, 0.1% SDS) containing HALT protease and phosphatase inhibitor cocktails (Pierce Biotechnologies). The samples were centrifuged at 15,000 rpm for 20 minutes and the supernatant collected. Protein concentrations were determined using the BCA reagent assay (Pierce).

### Western Blotting

Protein samples were separated by SDS-PAGE and blotted onto polyvinylidene difluoride (PVDF) membranes (Millipore Corporation, Billerica, MA, USA). Blots were blocked with 50 mM Tris HCl, pH 7.6, 0.15 M NaCl, 0.1% Tween-20 (TBST), 5% non fat dry milk and probed overnight with the relevant primary antibody diluted in blocking solution or in 5% BSA/TBST in the case of phospho-specific antibodies. Blots were then incubated for 1.5 hours with the appropriate horseradish peroxidase (HRP) conjugated secondary antibody (GE Healthcare Bio-Sciences Corporation, Piscataway, NJ, USA) diluted in blocking solution (1:5000-1:10,000) and bands visualized by ECL+ (GE Biosciences) after exposure to CL-Xposure film (Pierce) and/or imaged on a Kodak Image Station 4000R (Carestream Molecular Imaging, New Haven, CT, USA). Quantification was performed using Image Quant TL software (GE Biosciences). For reprobing, the membranes were treated with Re-Blot Plus strong stripping solution (Millipore) according to the manufacturer's instructions.

The primary antibodies utilized were rabbit polyclonal (1:1500 dilution; Novus Biologicals, Littleton, CO, USA) or monoclonal (Mab5382, 1:1000; Millipore) antibodies to HIF-1α, a monoclonal antibody to the cytoplasmic domain of N-cadherin (1:1000; BD Biosciences, San Jose, CA, USA), rabbit monoclonal antibodies to phospho-Akt (Ser473), total Akt, phospho-GSK-3β (Ser9), total GSK-3β, and the phospho-insulin receptor (all at 1:1500 dilutions; Cell Signaling, Beverly, MA, USA), as well as a mouse monoclonal antibody to the insulin receptor β (MAB 1139, 1:500; Millipore). Mouse monoclonal anti-actin antibody AC15 (1:500; Sigma Aldrich) or rabbit polyclonal anti β-tubulin (1:1500; Abcam, Cambridge, UK) antibodies were used as loading controls.

### Lentivirus infection

A lentiviral construct (2 μg) containing human Psen1 cloned in vector pReceiver-LV31 (Z 0049, Genecopeia, Rockville, MD, USA) was transfected with 10 μg of the packaging plasmids pLV-PK-FIV and pLV-PK-VSG (Genecopeia) into 293Ta cells using the Lipofectamine reagent (Invitrogen). Forty-eight hours post-tranfection the culture supernatant containing the recombinant lentiviruses was centrifuged for 10 minutes at 1500 rpm to clear debris and filtered through a 0.45 μm PVDF filter. To infect fibroblasts 1 ml of the lentiviral supernatant containing 8 μg/ml of polybrene was added (multiplicity of infection = 10). After overnight incubation, the viral supernatant was replaced with DMEM containing 20% fetal calf serum and cultured for 48 h. The cells were then treated with cobalt chloride and analyzed for HIF-1α as described above.

### Co-immunoprecipitations

An expression ready plasmid for mouse HIF-1α (clone ID 4019056) was obtained from Open Biosystems (Huntsville, AL, USA) and for human Psen1 (clone sc125532) from Origene (Rockville, MD, USA). Psen1 and HIF-1α expression plasmids (3 μg each) or empty vector were transfected singly or together into HEK293 cells using the Fugene 6 reagent as recommended by the manufacturer (Roche, Indianapolis, IN, USA). After 48 hours cells were treated for 1 hour with 100 μM MG132 and harvested. The cells were lysed in an IP buffer consisting of 50 mM Tris HCl pH 7.4, 150 mM NaCl, 1 mM EDTA, 0.5% NP-40 supplemented with a protease inhibitor cocktail (Halt, Pierce) and phosphatase inhibitor cocktails I and II (Sigma Aldrich). Lysates (500-750 μg protein) were pre-cleared with protein A/G (Santa Cruz Biotechnology, Santa Cruz, CA, USA) for 2 hours and then incubated overnight at 4°C with one of the following antibodies: rabbit anti-Psen1 NTF (1 μg; Santa Cruz), mouse monoclonal antibody NT.1 which recognizes the NTF of human Psen1 (3 μg, gift of Dr. Paul Mathews, Nathan Kline Institute, Orangeburg, NY, USA), a mouse monoclonal anti-Psen1 loop antibody (2 μg, MAB5232 Millipore), a mouse monoclonal anti-HIF-1α antibody (3 μg, clone ESEE 122, Novus) or rabbit IgG (Santa Cruz). 20 μl of protein A/G was then added for 2 hours at 4°C and the samples were washed 4 times (20 min each) with IP buffer. SDS-PAGE buffer was added and samples were boiled or for Psen1 blots heated at 50°C for 10 minutes and analyzed by Western blot using the antibodies described as well as the mouse monoclonal antibody 33B10 (1:3,000, gift of Dr. Nikolaos Robakis, Mount Sinai School of Medicine, New York, NY, USA) which recognizes both the human and mouse Psen1 CTF. Clean-Blot IP HRP detection reagent (Pierce) was used to minimize reactivity with denatured IgG in co-immunoprecipitations when the blots were probed with rabbit polyclonal antibodies. For co-immunoprecipitation of endogenous HIF-1α in *Psen1+/+ *fibroblasts, cells were treated with 100 μM CoCl_2 _and 10 μM MG132 for 4 hours and then immunoprecipitations were performed with anti-Psen1 antibodies as described above.

### Quantitation of mRNA expression by real time quantitative PCR

Total RNA was isolated from cultured cells using the Ribopure kit (Ambion, Austin, TX, USA). Residual genomic DNA was removed using the DNA-free kit (Ambion) and cDNA was synthesized from 0.8 μg of total RNA using the High Capacity cDNA Reverse Transcription Kit (Applied Biosystems, Foster City, CA, USA). Real-time quantitative polymerase chain reaction (qPCR) was performed using an ABI Prism 7500 or 7700 Sequence Detector and TaqMan FAM/MGB gene-specific fluorogenic assays mostly as previously described [88]. Pre-designed TaqMan Gene Expression assays (probe and primer mix) for all target and control genes were purchased from Applied Biosystems (Mm00437304_m1 VegfA, Mm00441480 Glut-1, Mm00468875_m1 HIF-1a, Mm00446953_m1 Gusb and Mm02342429_m1 Ppia). Each 20 μl reaction contained 5 μl of the relevant cDNA (diluted 25 times in H_2_O), 1 μl of a specific TaqMan assay, and 10 μl of the 2×PCR Universal Master Mix (Applied Biosystems). The thermal cycling program consisted of 2 min at 50°C, 10 min at 95°C, followed by 40 cycles of 15 sec at 95°C and 1 min at 60°C. Only one cDNA was amplified in each qPCR (monoplex). The reactions were run in triplicate for each sample. Relative expression values of target genes were calculated using the 2^DDCt ^method [89], with the amount of target normalized to the geometric mean of the expression values for the endogenous control genes peptidylprolyl isomerase A (Ppia) and Glucuronidase beta (Gusb) relative to a calibrator (generated by pooling all the samples).

### Statistical procedures

All data are presented as mean ± the standard error of the mean (S.E.M.). Statistical comparisons were made using unpaired t-tests (Student's t if the variances did not differ significantly, p > 0.05, by Levene's test; otherwise the Welch correction for unequal variances) or one-way analysis of variance (ANOVA) with Dunnett's post-test. Statistical tests were performed using the program GraphPad Prism 5.0 (GraphPad Software, San Diego, CA, USA) or SPSS 16.0 (SPSS, Chicago, IL, USA).

## Abbreviations

Aβ: β-amyloid; APP: amyloid precursor protein; AD: Alzheimer's disease; BAPTA-AM: 1,2-bis (o-aminophenoxy)ethane-N,N,N',N'-tetraacetic acid tetrakis (acetoxymethylester); CTF: C-terminal fragment; DMEM: Dulbecco's Modified Eagle's Medium; FAD: familial Alzheimer's disease; Glut-1: glucose transporter-1; GSK-3β: glycogen synthase kinase 3β; Gusb: glucuronidase beta; HIF-1α: hypoxia inducible factor-1α; HRP: horseradish peroxidase; IGF-1: insulin like growth factor-1; IP: immunoprecipitation; IR: insulin receptor; MAP kinase: mitogen activated protein kinase; NTF: N-terminal fragment; PAC: P1 bacteriophage artificial chromosome; PI3K: phosphatidylinositol 3-kinase; Ppia: peptidylprolyl isomerase A; Psen1: presenilin-1; Psen2: presenilin-2; PVDF: polyvinylidene difluoride; qPCR: quantitative polymerase chain reaction; PHDs: prolyl hydroxylases; SDS-PAGE: sodium dodecyl sulfate polyacrylamide gel electrophoresis; Vegf: vascular endothelial growth factor.

## Competing interests

The authors declare that they have no competing interests.

## Authors' contributions

RDG participated in the design, execution and data analysis of all the experiments as well as participated in the manuscript writing; MAGS participated in the experimental design as well as generation and maintenance of the fibroblast cell lines and manuscript writing; SP provided expert advice and assistance for the qPCR experiments including data analysis; GAE participated in the experimental design, data analysis and manuscript writing. All authors read and approved the final manuscript.
